# A Novel Multisensor Traffic State Assessment System Based on Incomplete Data

**DOI:** 10.1155/2014/532602

**Published:** 2014-08-04

**Authors:** Yiliang Zeng, Jinhui Lan, Bin Ran, Yaoliang Jiang

**Affiliations:** ^1^Department of Instrument Science and Technology, School of Automation and Electrical Engineering, University of Science and Technology Beijing, Beijing 100083, China; ^2^Department of Civil and Environmental Engineering, University of Wisconsin-Madison, Madison, WI 53706, USA

## Abstract

A novel multisensor system with incomplete data is presented for traffic state assessment. The system comprises probe vehicle detection sensors, fixed detection sensors, and traffic state assessment algorithm. First of all, the validity checking of the traffic flow data is taken as preprocessing of this method. And then a new method based on the history data information is proposed to fuse and recover the incomplete data. According to the characteristics of space complementary of data based on the probe vehicle detector and fixed detector, a fusion model of space matching is presented to estimate the mean travel speed of the road. Finally, the traffic flow data include flow, speed and, occupancy rate, which are detected between Beijing Deshengmen bridge and Drum Tower bridge, are fused to assess the traffic state of the road by using the fusion decision model of rough sets and cloud. The accuracy of experiment result can reach more than 98%, and the result is in accordance with the actual road traffic state. This system is effective to assess traffic state, and it is suitable for the urban intelligent transportation system.

## 1. Introduction

With the rapid development of the urbanization, the motor vehicle ownership and the road traffic flow are rapidly increasing, and the traffic congestion has become a common problem in the world [1]. Therefore, an accurate and scientific assessment of the current traffic state can provide the basis for traffic guidance systems and traffic control systems, optimize traffic management program, and reduce the incidence of traffic congestion. It is an important part to maximize the social and economic benefits of transportation resources [2]. More and more different types of vehicle detectors, such as coil detectors [3, 4], probe vehicles [5, 6], microwave detectors, and videos [7, 8], are employed to collect traffic information with the rapid development of sensor technology.

Many researchers have attempted to develop more efficient assessment models in order to obtain better results. Bachmann et al. [9] investigated the multisensor data fusion-based estimation techniques to fuse data from loop detectors and probe vehicles to accurately estimate freeway traffic speed. Bachmann et al. [10] studied the fusion techniques with Bluetooth and loop detector to improve the accuracy of traffic speed estimates. Berkow et al. [11] used the traffic signal and probe vehicles data to estimate the real-time transit location data and online implementation of arterial travel time information. Guo et al. [12] used Kalman filter approach to estimate the speed with single loop detector measurements under congested conditions. Kong et al. [13] proposed a fusion-based system composed of real-time traffic state surveillance, which can realize the real-time traffic state estimation with over 10,000 bidirectional road sections. In order to achieve the estimation of the state of the road network traffic, Kong et al. [14] also combined Kalman filter with evidence theory as a fusion platform and estimated the speed of the road network based on traffic wavelet theory. El Bantli [15] proposed the optimal linear estimation and weighted least squares method theoretically based on the incomplete traffic data, and the method was applied to estimate the road travel time. Klein et al. [16] used the data fusion and D-S theory to the decision support system of advanced traffic management. Li and McDonald [17] put forward a method of link travel time estimation by using a single GPS equipped on probe vehicle. Cheu et al. [18] put forward a fusion model based on neural network and test its effects by using simulation data. Smith and Conklin [19] used the local lane distribution patterns to estimate missing data values from traffic monitoring systems. The methodology used time-of-day lane distribution patterns at a particular location to estimate missing detector data and the results of this methodology showed that the error ranged from 6% to 8%. Chen et al. [20] proposed a method using historical data to detect bad data samples and impute them into missing or bad samples, and it gave a better estimate than previous methods. Treiber et al. [21] presented an advanced interpolation method for estimating smooth spatiotemporal profiles of local highway traffic variables such as flow, speed, and density, to fuse the traffic data and get the dynamic traffic information. Sumner [22] used fuzzy logic to fuse the detected traffic data information, quantified traffic conditions, and made a comprehensive assessment of the traffic state. Chang [23] applied the neural network to Brainmaker project, which made the current traffic state and historical information for pattern matching, and it improved the effect of computer traffic monitoring and automatic incident detection.

The traditional methods of urban road traffic state assessment are usually based on complete data obtained by the detection sensors. However, during the whole process of data acquisition, transmission, and processing, there are some factors, as follows, that cause incomplete data [24, 25]:

- the error installation and correction of the sensors;
- under the influence of abnormal weather or environment, which cause the occasional data exceptions or data missing;
- abnormal work of detection sensors;
- hardware or software failure of traffic management center system;
- communication interrupt between traffic detection sensors, regional controller, and traffic management center;
- no enough evaluation and sustainability maintenance of system.

These factors have a great impact on the effective and accurate assessment of the traffic state. These incomplete data are often manifested as irregular data collection, great data acquisition error, data missing, and so on [26, 27]. Texas Transportation Institute (TTI) showed that the complete rate range of traffic management system is from 16% to 93%, and the average value is 67% [28]. It means that the incomplete data is one of the outstanding problems existing in the traffic management system. Therefore, improving the effectiveness and completeness of traffic flow data and making road traffic running state assessment results more reasonable and accurate have an extremely important significance for the development of urban intelligent transportation.

In this paper, a new multisensor traffic state assessment system is developed. This system can obtain traffic data, which may include incomplete data, by coil detection sensors, microwave detection sensors, and probe vehicle detection sensors. This data is processed by a novel algorithm based on the fusion decision model of rough sets and cloud. The algorithm first checks the data validity. After the preprocessing, the selected incomplete traffic data are fused and recovered using the history information. Then, a space-matching fusion model is proposed to estimate the average travel speed. Finally, a fusion decision model of rough sets and cloud is presented to assess the traffic state using the flow, speed. and occupancy, which acquired form of the multisensors. Experimental results show that our system is suitable for traffic state assessment with incomplete traffic data.

## 2. Multisensor Traffic State Assessment System

In view of the existing problem that the traffic flow data is incomplete, a method of traffic state assessment based on multisensor is proposed.

**Figure 1:**
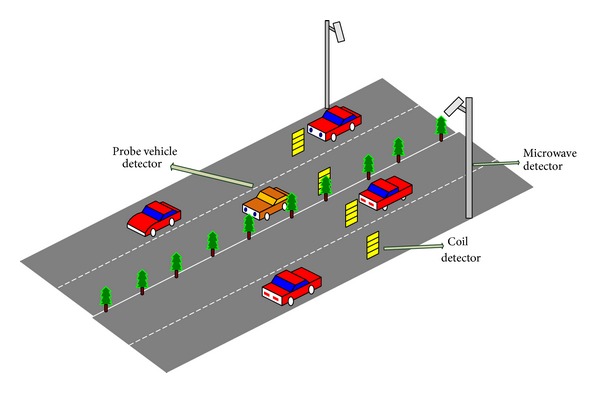
Testing environment of traffic flow data.

The system obtains the traffic data to get the multiple source information by the fixed detectors (coil detection sensors and microwave detection sensors) and the probe vehicle detector (floating car detection sensors). The testing environment of traffic flow data and the main system elements are shown in Figure 1.

The main three sensors of this system are as follows:

### 2.1. Coil Detection Sensors

Generally, coil detection sensors with square shape are laid under the roads as shown in Figure 1. When vehicles pass from these coil sensors, the inductance value of coil loop will be changed, which cause the change of frequency. And the detection sensors use this change to judge whether there are cars that pass the sensor or not. This kind of sensors can detect the traffic flow, speed, queue length, and other traffic parameters. The advantages of this sensor are low cost, high reliability, and high detection precision. However, when the distance between vehicles is less than 3 meters, the detection precision will be greatly reduced due to the magnetic field interference.

### 2.2. Microwave Detection Sensors

Microwave detectors shown in Figure 1 are sensors using microwave transmission form to detect traffic data. They send microwave in the test roads and detect the traffic parameter by calculating the receiving frequency and receiving time. Microwave detection sensors can detect traffic information, such as flow, occupancy, speed, and direction. This kind of sensors can adapt to all kinds of bad weather and have strong anti-interference ability, but it will greatly reduce the detection accuracy, while the vehicle speed is relatively slow.

### 2.3. Probe Vehicle Detection Sensors

Global position system (GPS) is used as the probe vehicle detection sensor to collect the speed data of vehicles, which can describe the change of current traffic flow. It can offer the traffic flow and instantaneous speed directly and provides travel time and travel speed indirectly. GPS data have a strong continuity and the acquisition range is extensive. However, the probe vehicle detection precision is affected by the GPS positioning accuracy, and data communication is susceptible to electromagnetic interference.

**Figure 2:**
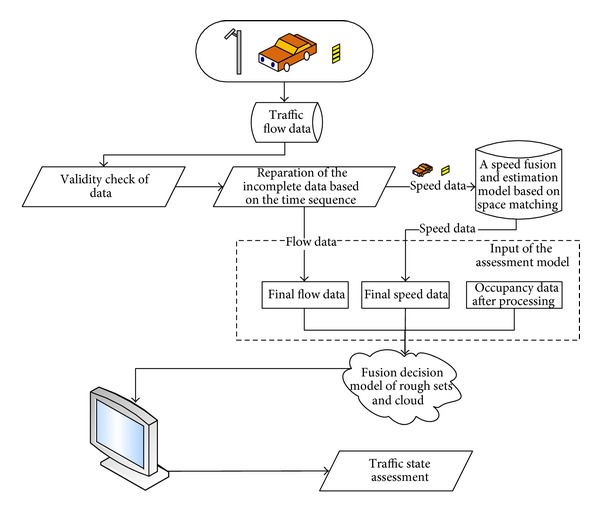
Overall flow chart of the proposed assessment method.

The assessment method is also one of the important elements in our multisensor traffic assessment system. And the overall flow chart of the assessment method is as shown in Figure 2.

## 3. Validity Check of Multisensor Data

The purpose of validity check is to screen the incomplete data out of traffic flow information and reduce the interference during the process of traffic state assessment. Three parameters of traffic data flow and the mechanism of traffic flow are used to adapt to the validity check of different types of incomplete data.

The method mainly includes the following four steps.

**Step 1 (basic data screening).**

Before macrodata screening, these data need to determine whether it contains a negative or missing data [21]. Three basic traffic parameters, traffic flow *q*, speed *v*, and occupation *o*, are considered. Through analyzing the relation of three parameters, the incorrect data can be screened. The approach is listed in Table 1.

**Step 2 (threshold inspection).**

The threshold test determines the upper and lower threshold of single information based on the statistical data. If the test value is not in the range of the upper and lower threshold, it can be considered to be erroneous data. Taking a lane, for example, there is a maximum limit value of flow and the minimum value is 0; at the same time, the maximum value of occupancy is 100% and the minimum is 0%.

**Step 3 (mechanism inspection of traffic flow).**

The theory of the mechanism inspection is mainly according to the basic characteristic of the traffic flow and the functional relation between the three parameters of the traffic flow. If the data does not conform to the inherent rule of traffic flow theory, this data set can be considered wrong and it should be deleted or recovered.

**Step 4 (abnormal inspection).**

Under normal traffic conditions, the change in the network traffic flow is a stationary random process. And the amplitudes of traffic data should be within a certain range of change. However, when a traffic incident occurs, there goes a large deviation. This paper uses the mean value ${\overset{-}{q}}_{t}$ and variance *σ*_*t*_ of previous *n* data of *t* moment to identify the fault data. That when ${\overset{-}{q}}_{t}-2\sigma \leq q_{t}\leq {\overset{-}{q}}_{t}+2\sigma$ is satisfied, the data is normal or is abnormal [22].

**Figure 3:**
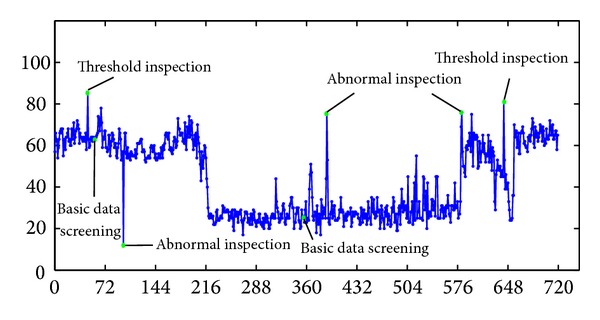
Data screening results.

The above four steps can almost deal with all possible data error. Take an example of traffic flow data, the fault data is filtered after the validity check and the result is shown as in Figure 3.

## 4. Traffic State Assessment Method

The traffic state assessment method includes three stages. First, restore the incomplete traffic flow data. Second, fuse and estimate the speed value. Third, build fusion decision model.

### 4.1. Restoration of Incomplete Traffic Flow Data

The traditional restoration algorithm based on incomplete data includes linear interpolation algorithm, historical trend restoration algorithm, restoration method based on the spatial correlation, and restoration method based on the BP neural network [23]. The advantages and disadvantages of methods are shown in Table 2.

Due to the heavy traffic on the road, the traffic flow data have small fluctuation and show the obvious time correlation obviously. So the historical data should be used for fusion estimation. In this paper a traffic data restoring algorithm based on the generation of area geometry, which specializes in the analysis of history traffic flow data and the connection between the area geometric formed by the adjacent historical data and the present moment data, is proposed.

The area of the geometric region formed by historical data can reflect the changing trends and the oscillation range of traffic flow data. So we make full use of the area to restore the present moment incomplete traffic flow data. The volatility of the traffic flow data can be shown by the recovered data.

**Figure 4:**
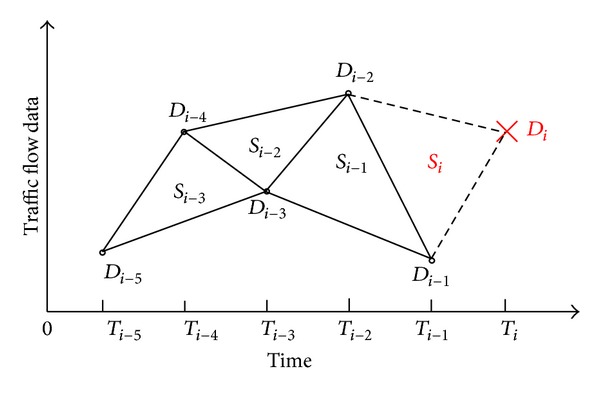
Sketch map of triangle area geometry by traffic flow data.

Take the example of flow. As shown in Figure 4, the flow data *D*_*i*−5_, *D*_*i*−4_, *D*_*i*−3_, *D*_*i*−2_, *D*_*i*−1_ are obtained by the traffic detector, respectively, at the moment *T*_*i*−5_, *T*_*i*−4_, *T*_*i*−3_, *T*_*i*−2_, and *T*_*i*−1_. Due to the fault of sensor or transmission, the flow data *D*_*i*_ at the moment *T*_*i*_ is incomplete. The area of the triangle is defined as *S*_*i*−1_, and it reflects the nonlinear degree of *D*_*i*−3_, *D*_*i*−2_, *D*_*i*−1_. When *S*_*i*−1_ is large, the oscillation amplitude of data *D*_*i*−3_, *D*_*i*−2_, *D*_*i*−1_ is increasing. And when *S*_*i*−1_ is 0, it indicates that the data *D*_*i*−3_, *D*_*i*−2_, *D*_*i*−1_ is changed in liner by time. There is a correlation between the data *D*_*i*_ and the historical data and their nonlinear trend. So that *S*_*i*_, the area *S*_*i*_ of  Δ*D*_*i*−2_ *D*_*i*−1_ *D*_*i*_, is connected with *S*_*i*−1_. In order to make the restored value more reliable, the area *S*_*i*−2_ of Δ*D*_*i*−4_ *D*_*i*−3_ *D*_*i*−2_ and the area *S*_*i*−3_ of  Δ*D*_*i*−5_ *D*_*i*−4_ *D*_*i*−3_ are taken into account. The three triangles are given different weights to determine the area *S*_*i*_ of  Δ*D*_*i*−2_ *D*_*i*−1_ *D*_*i*_ finally. The function for calculating *S*_*i*_ is defined as the following formula:

(1) $$\begin{array}{c} S_{i}=\omega_{1}S_{i-1}+\omega_{2}S_{i-2}+\omega_{3}S_{i-3}, \end{array}$$

where *ω*_1_, *ω*_2_, and *ω*_3_ are the weights of  *S*_*i*−1_, *S*_*i*−2_, and *S*_*i*−3_. And then the method is used to get the weights.

Define

(2) $$\begin{array}{c} \eta_{1}=\frac{S_{i-3}}{S_{i-4}},\;\;\eta_{2}=\frac{S_{i-2}}{S_{i-3}},\;\;\eta_{3}=\frac{S_{i-1}}{S_{i-2}}, \end{array}$$

where *S*_*i*−4_ is the area of Δ*D*_*i*−6_ *D*_*i*−5_ *D*_*i*−4_. And *η*_*j*_  (*j* = 1,2, 3) is the intermediate variable used to calculate the weights *ω*_*j*_.

And define

(3) $$\begin{array}{c} \omega_{j}=\frac{\eta_{j}}{\eta_{1}+\eta_{2}+\eta_{3}},\;\left(j=1,2,3\right). \end{array}$$

Therefore, if the geometric area constituted by the incomplete data and the last two neighboring data is fixed, the incomplete data can be fixed soon.

We assume that there are two units between the adjacent moments. Then the height of the triangle Δ*D*_*i*−2_ *D*_*i*−1_ *D*_*i*_ is computed by the following formula:

(4) $$\begin{array}{c} h=\frac{2S_{i}}{D_{i-1}D_{i-2}}=\frac{2S_{i}}{\sqrt{4+{\left(D_{i-1}-D_{i-2}\right)}^{2}}}. \end{array}$$

And the linear equation of *D*_*i*−2_ *D*_*i*−1_ is

(5) $$\begin{array}{c} \frac{D_{i-2}-D_{i-1}}{2}t+q-D_{i-2}+\frac{T_{i-2}\left(D_{i-1}-D_{i-2}\right)}{2}=0. \end{array}$$

According to Formulas (4) and (5), we can get two traffic flow data values at *T*_*i*_ moment and these are *D*_*i*_ and *D*_*i*_′ (defining *D*_*i*_ > *D*_*i*_′)

(6) $$\begin{array}{c} D_{i}=2D_{i-1}-D_{i-2}+S_{i},\\ D_{i}^{\prime }=2D_{i-1}-D_{i-2}-S_{i}. \end{array}$$

**Figure 5:**
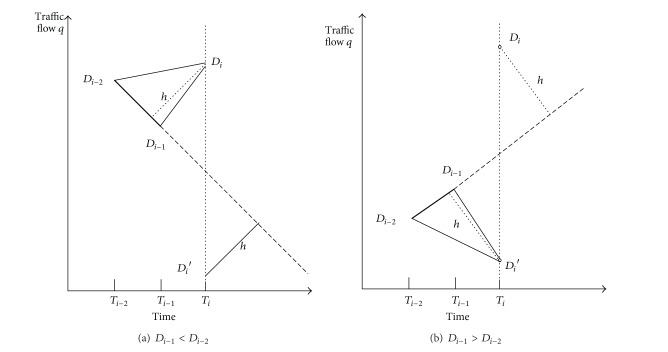
Solving process of the flow value.

The solving process is shown in Figure 5. It can be figured that the flow value of  *T*_*i*_ moment is equal to *D*_*i*_, when *D*_*i*−1_ < *D*_*i*−2_, and equal to *D*_*i*_′ when *D*_*i*−1_ > *D*_*i*−2_. This ensures that the restored data for *T*_*i*_ moment reflects both the historical data trends and the oscillation amplitude.

### 4.2. Fusion and Estimation of Speed Based on Space Matching

**Figure 6:**
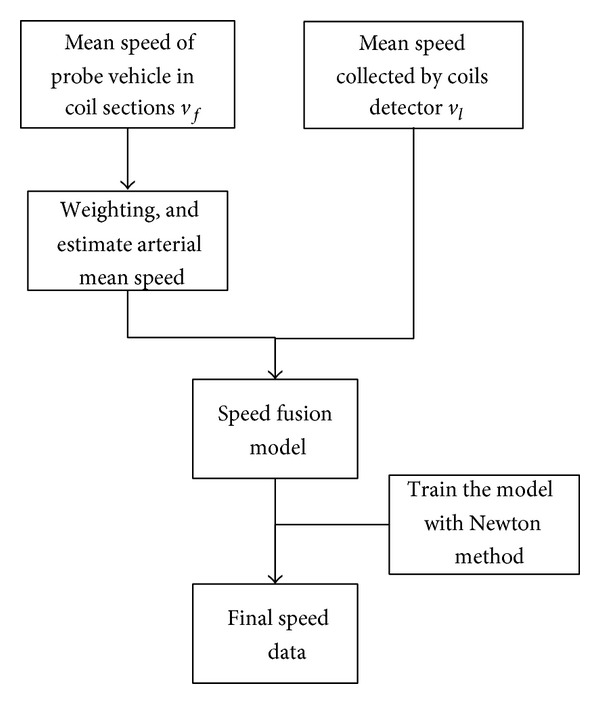
Flow chart of speed fusion and estimation based on space matching.

In order to improve the effectiveness and accuracy of traffic flow data, a fusion and estimation model of speed based on space matching is proposed in this paper. This method uses the mean speed information from the probe vehicle detector and the coil detector, sets up the fusion model of road speed, and trains the weights and deviation of the model by Newton method, to obtain the final speed data. The flow chart is shown in Figure 6.

#### 4.2.1. Speed Fusion and Estimation Model

**Figure 7:**
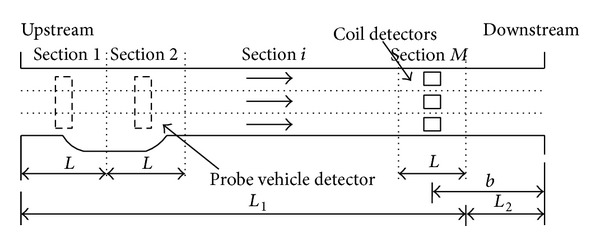
Speed estimation model of urban road.

The speed fusion and estimation model based on probe vehicle detector and coil detector [29] is built as shown in Figure 7.

In the model, the whole road is divided into upstream and downstream, which is expressed with *L*_1_ and *L*_2_, respectively. On the downstream side of the road, because of the influence of the traffic lights, traffic will be lined up; so it is unable to provide effective information for sections of the mean travel speed; so this paper selects the upstream road sections as the research object to estimate the mean speed. The upstream of the road is divided into *M* sections of equal length, the *M*th is close to the downstream of whole road, and the coil is placed on the *M*th section, so that we can get the parameters of the vehicles such as flow, speed, and occupancy through the cross section. There is no fixed detector in sections 1 to *M* − 1, and the dotted boxes represent the spots of the cross section. The data come from the probe vehicle detector, and this model is mainly used to access the speed data of probe vehicle detector. In this paper, the data of probe vehicle detector is regarded as coil detector.

#### 4.2.2. Speed Fusion Method

Since the probe vehicle detector is a part of the traffic participants, and on the other hand, the coil detector can only collect the spot speed, so it cannot estimate the mean speed very well. For these reasons, it is necessary to make space-matching data of the probe vehicle detector and coils. In other words, eliminate the difference between the data of probe vehicle detectors and data of coils with data correction.

According to Figure 7, the mean speed in every section can affect arterial mean speed; so it can be estimated arterial mean speed through the weight sum of mean speed in every section

(7) $$\begin{array}{c} \overset{-}{v}=\overset{M}{\underset{i=1}{\sum }}w_{i}{\overset{-}{v}}_{i}+b, \end{array}$$

where $\overset{-}{v}$ is the arterial mean speed (km/h), ${\overset{-}{v}}_{i}$ is the mean speed of  *i*th section (km/h), and *w*_*i*_ is the weight of the corresponding section. *w*_*i*_ ∈ [0,1].  *b* is the deviation, which is used to correct fusion result. So the function of total error is

(8) $$\begin{array}{c} \text{En}=\frac{1}{2}\overset{N}{\underset{i=1}{\sum }}{\left[\overset{-}{v}(i)-v(i)\right]}^{2}, \end{array}$$

where $\overset{-}{v}(i)$ is the estimated mean speed of the *i*th sample and *v*(*i*) is the actual mean speed of  *i*th sample. *N* is the total number of sample data.

In order to find weight and deviation when getting the minimal total error, it needs to train Fusion model. The weight is trained by Newton method, which is a fast optimal method based on quadratic's Taylor series. Newton method is defined as

(9) $$\begin{array}{c} x_{k+1}=x_{k}-A_{k}^{-1}g_{k}, \end{array}$$

where *x*_*k*+1_ is *k* + 1th weight or deviation, *x*_*k*_ is previous weight or deviation, *g*_*k*_ is coefficient of variable, and *A*_*k*_^−1^ is Hessian matrix which is obtained from error performance function in the current weights and threshold value.

The basic idea of Newton method is that with a quadratic function locally approximate *f*(*x*) at first and then find minimum of approximated function. The Hessian function can be expressed as [30]

(10) $$\begin{array}{c} \nabla^{2}f\left(x\right)=2J^{T}\left(x\right)J\left(x\right)+2S\left(x\right), \end{array}$$

where *J*(*x*) is Jacobean matrix:

(11) $$\begin{array}{c} S\left(x\right)=\overset{N}{\underset{i=1}{\sum }}v_{i}\left(x\right)\nabla^{2}v_{i}\left(x\right), \end{array}$$

where *v*_*i*_(*x*) is the error vector. When *S*(*x*) is small, Hessian matrix is approximately expressed as

(12) $$\begin{array}{c} \nabla^{2}f\left(x\right)≅2J^{T}\left(x\right)J\left(x\right). \end{array}$$

If *f*(*x*) is the form of (8), gradient can be expressed as follows:

(13) $$\begin{array}{c} \nabla f\left(x\right)=2J^{T}\left(x\right)v\left(x\right), \end{array}$$

where

(14) $$\begin{array}{c} J\left(x\right)=\left[\begin{array}{cccc} \frac{\partial v_{1}\left(x\right)}{\partial x_{1}} & \frac{\partial v_{1}\left(x\right)}{\partial x_{2}} & \cdots & \frac{\partial v_{1}\left(x\right)}{\partial x_{n}}\\ \frac{\partial v_{2}\left(x\right)}{\partial x_{1}} & \frac{\partial v_{2}\left(x\right)}{\partial x_{2}} & \cdots & \frac{\partial v_{2}\left(x\right)}{\partial x_{n}}\\ \vdots & \vdots & & \vdots \\ \frac{\partial v_{N}\left(x\right)}{\partial x_{1}} & \frac{\partial v_{N}\left(x\right)}{\partial x_{2}} & \cdots & \frac{\partial v_{N}\left(x\right)}{\partial x_{n}} \end{array}\right]. \end{array}$$

Making second derivative to formula (13), the *k*, *j*th element of result is that

(15) [∇2f(x)]k,j=∂2f(x)∂xk∂xj=2∑i=1N{∂vi(x)∂xk∂vi(x)∂xj+vi(x)∂2vi(x)∂xk∂xj}.

So Newton method is expressed as follows:

(16) $$\begin{array}{c} x_{k+1}=x_{k}-{\left[J^{T}(x_{k})J(x_{k})\right]}^{-1}J^{T}\left(x_{k}\right)v\left(x_{k}\right). \end{array}$$

The Newton method has fast convergence speed and always can be found minimum of quadratic function in one step; so it can be used to train weight and deviation of fusion model. When data of probe vehicle and data of coils detector are fused, the fusion result can reduce training time and reduce the consumption of computer resource with this method. It also can guarantee real-time performance of fusion algorithm.

### 4.3. Fusion Decision Model of Rough Sets and Cloud

After the restoration and estimation, a fusion decision model of rough sets and cloud is presented in this paper to assess the road traffic state.

#### 4.3.1. Cloud Model Review

Assume *U* is the quantitative domain represented by an exact value and *C* is the qualitative concept of *U*. If quantitative value *x* is a random realization of concept *C*, and *x* ∈ *U*, therefore, *μ*(*x*)∈[0,1] which refers to the membership grade of *x* in *C*, is a random number with a stable tendency

(17) $$\begin{array}{c} \mu \left(x\right):U⟶\left[0,1\right],\;\forall x\in U,\;\;x⟶\mu \left(x\right). \end{array}$$

**Figure 8:**
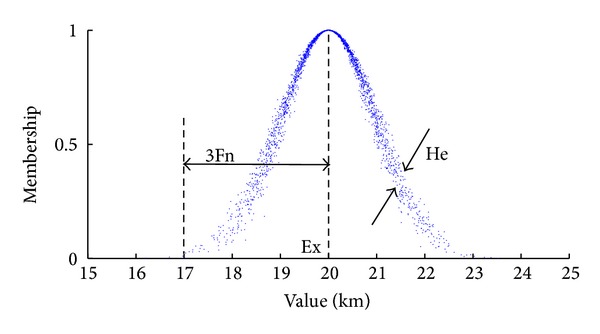
Digital features of cloud.

The distribution of  *x* in *U* is called cloud model, and *x* is called cloud drop, just as shown in Figure 8.

There are three digital features of cloud [31, 32]: expected value Ex, entropy En, and hyper entropy He. Ex is described as the center of the whole cloud drop in the domain *U*. It reflects the digital domain coordinates which has the most representative of the concept. En is the fuzzy measurement of the qualitative concept. It reflects the range that can be accepted by the language value in the digital domain. He is the degree of dispersion of the entropy En, which is the entropy of En. It reflects cohesion degree of the cloud drops.

If the membership grade *μ*(*x*) of *x* in *C* satisfies the following equation [33]:

(18) $$\begin{array}{c} \mu \left(x\right)=\exp\left[-\frac{{\left(x-\text{Ex}\right)}^{2}}{2{\left(\text{E}{\text{n}}^{\prime }\right)}^{2}}\right], \end{array}$$

where *x* ~ *N*(Ex, En^′2^) and En′ ~ *N*(En, He^2^), then the distribution of  *x* in *U* is called normal cloud [34].

#### 4.3.2. Cloud Generator Review

**Figure 9:**
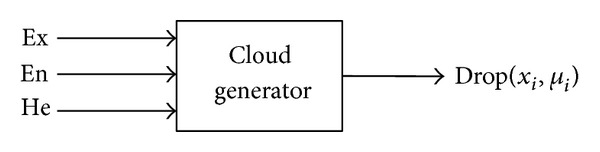
Forward cloud generator.

There are mainly two kinds of cloud generators, named forward cloud generator and backward cloud generator [35–37]. Forward cloud generator is described as the algorithm to generate a quantity of cloud drops drop(*x*_*i*_, *μ*_*i*_) of the normal cloud model by using the three digital characteristics (Ex, En, He), which is shown in Figure 9.

The forward cloud generator algorithm description is as follows.

**Step 1 .**

Generate a Gaussian random number En_*i*_′, with the expected value En and the standard deviations He^2^.

**Step 2 .**

Generate a Gaussian random number *x*_*i*_, with the expected value Ex and the standard deviations En_*i*_′.

**Step 3 .**

Make *x*_*i*_ be a detailed quantitative value of concept *A*, called the cloud droplets.

**Step 4 .**

Calculation formula (18).

Repeat Steps from 1 to 4 until producing *N* cloud droplets.

**Figure 10:**
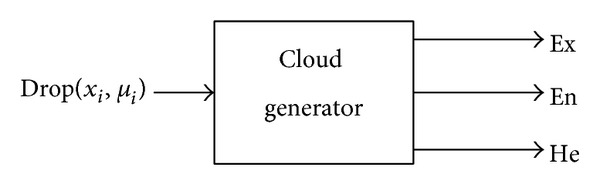
Back cloud generator.

And the backward cloud generator is the inverse process of the forward cloud generator; it transforms the given sample of data to the qualitative concept, with the expression by digital characteristics of the cloud {Ex, En, He}; it is a mapping from sample of data to concept, which is shown in Figure 10.

The back cloud generator algorithm description is as follows.

**Step 1 .**

Regard $\hat{\text{Ex}}=\left(1/n\right)\sum_{i=1}^{m}x_{i}$ as the estimated value of Ex.

**Step 2 .**

Remove the cloud droplets which *μ*_*i*_ > 0.999, remaining *m* cloud droplets.

**Step 3 .**

Work out En′ from $\text{E}{\text{n}}^{\prime }=\left|x-\hat{\text{Ex}}\right|/-\sqrt{2|\ln\mu_{i}|}$.

**Step 4 .**

According to $\hat{\text{En}}=\left(1/m\right)\sum_{i=1}^{m}\text{E}{\text{n}}_{i}^{\prime }$, acquire $\hat{\text{En}}$, the estimated value of En.

**Step 5 .**

According to $\hat{\text{He}}=\sqrt{\left(1/(m-1)\right)\sum_{i=1}^{m}{\left({\text{En}}_{i}^{\prime }-\hat{\text{En}}\right)}^{2}}$, acquire $\hat{\text{He}}$, the estimated value of He.

#### 4.3.3. Rough Set Theory Review

The main idea of rough set theory [38] is to divide the given space according to the equivalence relation; at the same time the equivalence property of knowledge is guaranteed. Attribute reduction is an important content of rough set; it deletes the redundant or unimportant condition attributes and attribute values under the condition that keeping the constant ability of classification, and then get the rules of the condition attribute relative to decision attribute decision.

The method is simple and does not need any priori information; so it can be applied into the generation of fusion decision rules. Because of its objectivity uncertainty description of the problem, it is well applied in traffic state assessment.

#### 4.3.4. Proposed Fusion Decision Model

When using the rough set theory to the analysis of the actual data and knowledge, each attribute value of the decision table must be discrete, and though there exists fluctuation in traffic flow data, in the local scope it has certain continuity. So in this paper, we use the cloud model to realize the discretization of traffic flow data.

The fusion decision model of rough sets and cloud is mainly based on cloud model theory. The algorithm steps are as follows.

**Step 1 .**

For multiple parameters of traffic detector, select the qualitative concept, respectively, and determine the scope of its quantitative value.

**Step 2 .**

According to the cloud model theory, generate a different qualitative concept of cloud, respectively, and make the continuous values of traffic flow data discrete.

**Step 3 .**

Regard the discrete traffic parameters of the samples as condition attributes, obtain the status value of every moment as decision attribute according to the expert system, and establish a decision table.

**Step 4 .**

Delete duplicate objects in a decision table.

**Step 5 .**

Calculate each of the importance degree of condition attributes for decision attribute and delete the condition attribute whose important degrees are 0.

**Step 6 .**

According to the knowledge reduction method of rough set, delete redundant condition attributes.

**Step 7 .**

Delete the redundant attribute values for each object and obtain the final decision rules.

## 5. Experimentation and Results

### 5.1. Result of Data Restoration

**Figure 11:**
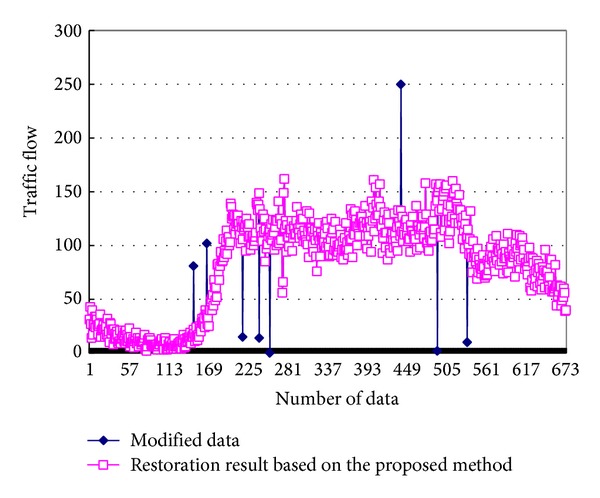
Data comparison before and after restoration.

The traffic flow data, which were acquired from the Beijing DeShengMen bridge to the Drum Tower in June 19, 2009, were taken as the original data. In order to test the effectiveness of the incomplete data restoration algorithm, the original data were modified and manufacture some incomplete data artificially. Then we used the proposed algorithm to deal with the incomplete data, and the result is shown in Figure 11.

Figure 11 shows that the 8 incomplete data points under this algorithm can effectively be detected accurately. To further illustrate the effectiveness of the algorithm, we compare with two other algorithms: the linear interpolation algorithm and the historical trend of restoration algorithm. And the result is shown in Table 3. Take number 158 as an example, the modified data means that we change the original data from 23 to 81. Compare with the relative error of different algorithms, the mean relative error of the proposed algorithm is 1.85%, while the historical trend restore algorithm is 14.78%, and the restoration of the linear interpolation algorithm is 11.90%. The effectiveness of the proposed algorithm in this paper is much better than other methods.

### 5.2. Result of Speed Fusion Experiments

**Figure 12:**
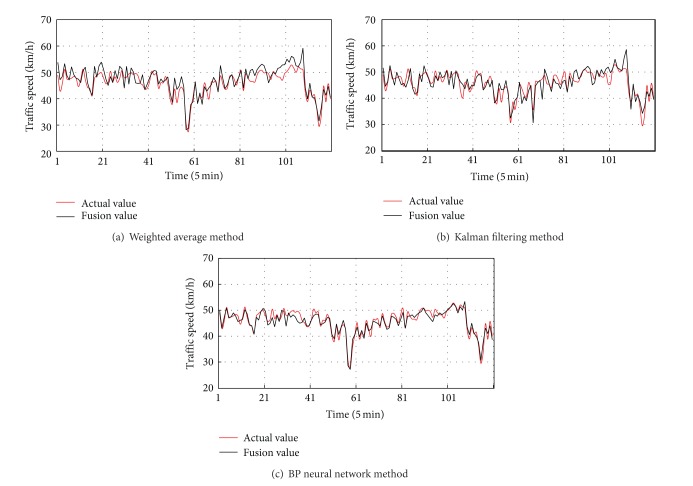
Results of the three methods.

In order to verify the reliability of the algorithm, weighted average method, Kalman filtering method, and BP neural network method have been taken into the experiment analysis. The specific analysis of experimental data with the three methods is shown in Figure 12.

**Figure 13:**
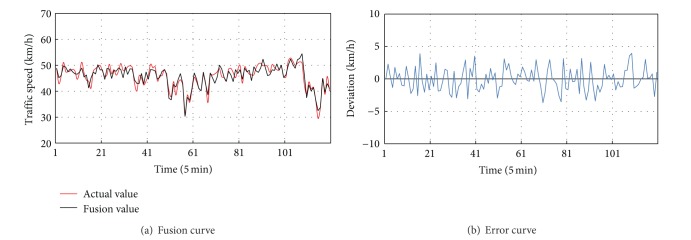
Fusion curve and error curve of space-matching fusion method.

After the match of data detected by probe vehicle detector, we extracted the speed in each section of the road, respectively, and take its average value as the input of the fusion model. Here the road is divided into six subsections and the data detected by coil detector is constant. The 60% of the data is taken as the training sample with the method of 10-fold cross validation, and the Newton method is used to determine the weight of each speed value. And then the remaining 40% data is tested with the steps as mentioned above. The result of the space-matching fusion method and error curve is shown in Figure 13.

We assess the strengths and weaknesses of these methods with these indicators such as mean absolute error (MAE), mean square error (MSE), mean absolute percentage error (MAPE), mean square percentage error (MSPE), and the maximum error (MAXERR (%)). The evaluation results are shown in Table 4.

Through observing the comparison results, we find that the evaluation result of space-matching fusion method is much better than the weighted average fusion method and the Kalman filtering method and much similar to the fusion effect of the neural network method. But the calculation neural network method is relatively complex. In conclusion, the method of fusion and estimation of speed based on space matching not only guarantees the timeliness but also improves the reliability and validity of data.

### 5.3. Analysis of Traffic State Assessment Result

Qualitative concepts of traffic flow parameters are given as follows: traffic flow = {very low, low, normal, high, very high}, speed = {very slow, slow, normal, fast, very fast}, and occupancy = {very low, low, normal, high, very high}; we use 0, 1, 2, 3, and 4 to represent the qualitative concept, respectively. And in Table 5, the threshold value of qualitative concepts of flow, speed, and occupancy is listed.

The cloud models *C*_*n*_(Ex_*n*_, En_*n*_, He_*n*_) are shown in Table 6.

Then, the collected traffic flow data and the cloud listed in Table 6 are substituted into (18), respectively, if *μ*_*k*_ is the maximum in *C*_*i*_  (*i* = 0,1,…, *n*), then traffic flow parameters value belongs to the cloud *k*.  Table 7 lists parts of the identification results traffic flow parameters based on cloud theory.

We can get the final decision rules based on rough set theory, which is shown in Table 8.

**Figure 14:**
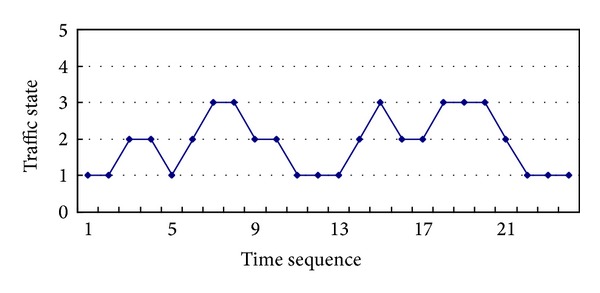
Results of assessment of traffic state.

Figure 14 lists the results of assessment of the traffic state. There are four states below: 1 represents the smooth traffic, 2 represents the slight congestion, 3 represents the moderately congestion, and 4 represents the overcrowded.

In order to explain the effectiveness of the algorithm better, we use crowded identification rate (IR) and false identification rate (FIR) to test the algorithm. The test result is shown in Table 9. It shows that the identification rate is over 98% and the misjudgment rate is low.

Experimental results show that the restoring algorithm based on self-adaptive generation of area geometry and the fusion and estimation model of speed based on space matching improve the completeness and effectiveness of the traffic flow data. The fusion decision model of rough sets and cloud can be used to assess the traffic state and achieve the desired results.

## 6. Conclusion

In this paper, a multisensor traffic state assessment system was developed. As the sensors usually acquire incomplete data of traffic data, our system provides a novel and robust algorithms to solve this problem. The results of the restoring algorithm based on self-adaptive generation of area geometry are comparatively consistent with the real data, and the mean relative error is only 1.85%, which improves the reliability of the data greatly. And with the speed fusion estimation model based on space matching, the estimation precision is above 90%, which improves the effectiveness and the accuracy of the speed data. Finally, the traffic state assessment based on the fusion decision model of rough sets and cloud is applied to the actual road traffic condition, and the evaluation accuracy is above 98%. The experiment results show that the proposed system is feasible, effective, and accurate, and it has great important significance to the development of the urban intelligent transportation system.

## Figures and Tables

**Table 1:** Basic data screening based on traffic parameters.

Number	Parameters	Validity judgement	Approach
1	*q* = 0, *o* = 0, *v* = 0	Missing data or true value	Next inspection
2	*q* ≠ 0, *o* = 0, *v* = 0	Error	Delete or restore
3	*q* = 0, *o* ≠ 0, *v* = 0	Error	Delete or restore
4	*q* = 0, *o* = 1, *v* = 0	Parking	Next inspection
5	*q* = 0, *o* = 0, *v* ≠ 0	Error	Delete or restore
6	*q* = 0, *o* ≠ 0, *v* ≠ 0	Error	Delete or restore
7	*q* ≠ 0, *o* ≠ 0, *v* = 0	Error	Delete or restore
8	*q* ≠ 0, *o* = 0, *v* ≠ 0	Undetermined	Next inspection
9	*q* ≠ 0, *o* ≠ 0, *v* ≠ 0	Undetermined	Next inspection

**Table 2:** Restoration methods comparation.

Restoration methods	Advantages	Disadvantages
Linear interpolation restoration algorithm	Simple calculation	The error is large and cannot be applied in practice
Historical trend restoration algorithm	Simple and easy	The data is smooth, and it is difficult to reflect the volatility of traffic data
Restoration method based on the spatial correlation of data	Improving the real time, reflecting the real traffic state accurately	Must know other lanes of traffic flow data in advance
Restoration method based on the BP neural network of data	High precision and good effect	Complex calculation and poor real time

**Table 3:** Comparation of the three methods.

Data Number	Data types							
	Original data	Modified data	Proposed algorithm		Historical trend of restoration algorithm		Linear interpolation algorithm	
			Result	Relative error (%)	Result	Relative error (%)	Result	Relative error (%)
158	23	81	21.67	5.78	14	39.13	16.5	30.43
179	35	102	34.54	1.31	36.25	3.57	31.5	10
232	105	15	105.78	0.74	106	0.95	119.5	13.80
257	148	14	148.78	0.53	107.75	27.20	136	8.11
273	114	0	115.35	1.18	108.5	4.82	110	3.51
471	133	250	132.4	0.45	110	17.29	111	16.54
526	143	2	141.58	0.99	113	20.98	148	3.50
572	102	10	105.92	3.84	97.5	4.41	111.5	9.31

Average				1.85		14.78		11.90

**Table 4:** Evaluation results of different speed fusion method.

	MAE	MSE	MAPE (%)	MSPE	MAXER	MAXERR (%)
Weighted average method	2.0550	0.2343	4.4993	0.0051	8.3000	16.3065
Kalman filtering method	2.0624	0.2375	4.6198	0.0054	7.8459	16.5525
BP neural network method	1.2000	0.1375	2.6683	0.0031	4.2000	11.9047
Proposed method	1.4803	0.1641	3.2637	0.0037	3.9529	10.2142

**Table 5:** Threshold value of qualitative concept.

Number	Flow		Speed (km/h)		Occupancy (%)	
	Min	Max	Min	Max	Min	Max
0	19	40	3.8	12	1.1	12
1	30	50	10	22	9	20
2	40	90	18	32	18	32
3	80	120	28	40	30	55
4	110	150	35	53.5	50	73.1

**Table 6:** Different cloud mode.

Number	Flow	Speed	Occupation
0	C_0_(19, 3.5, 0.1)	C_0_(3.8, 1.37, 0.1)	C_0_(1.1, 1.82, 0.01)
1	C_1_(40, 3.33, 0.1)	C_1_(16, 2, 0.1)	C_1_(14.5, 1.88, 0.1)
2	C_2_(65, 8.33, 0.1)	C_2_(25, 2.33, 0.1)	C_2_(25, 2.33, 0.1)
3	C_3_(100, 6.67, 0.1)	C_3_(34, 2, 0.1)	C_3_(42.5, 4.17, 0.1)
4	C_4_(150, 6.67, 0.1)	C_4_(53.5, 3.08, 0.1)	C_4_(73.1, 3.85, 0.1)

**Table 7:** Identification results of traffic flow parameter.

Number	Flow	Speed	Occupation	Congestion level
1	Very low	Very fast	Very low	Free
2	Low	Very fast	Very low	Free
3	Very high	Fast	High	General
4	Normal	Normal	Very low	General
	⋮			⋮
129	High	Slow	Normal	Moderate
130	High	Slow	Low	Moderate
131	Normal	Very slow	High	Serious
132	High	Very slow	High	Serious

**Table 8:** Final decision rules.

Number	Conditional attribute			Traffic state
	Queue delay	Flow	Speed	
1	—	—	Very fast	Free
2	—	Normal	Fast	
3	No delay	High	—	

4	Accept	High	Fast	General congest
5	—	Very high	Fast	
6	—	Normal	Normal	
7	Accept	High	Normal	

8	Can accept	—	Normal	Moderate congest
9	—	Very high	Normal	
10	—	Normal	Slow	
11	Accept	—	Slow	
12	Can accept	High	Slow	

13	Cannot accept	—	—	Serious congest
14	—	Very high	Slow	
15	—	—	Very slow	

**Table 9:** Evaluation results of the method.

Indicator	IR	FIR
The result	98.26%	0.035%
